# Outcomes of Colonic and Gastric Tube Transplants after Caustic Esophageal Burn in Children: A 33-Year Review

**DOI:** 10.3390/jcm13164689

**Published:** 2024-08-09

**Authors:** Michaël de Sousa Amaral, Sabine Vasseur Maurer, Olivier Reinberg, Natalie Divjak, Anthony de Buys Roessingh

**Affiliations:** Department of Pediatric Surgery, University Hospital Center of the Canton of Vaud (CHUV), 1011 Lausanne, Switzerland

**Keywords:** esophagus, esophageal replacement, caustic lesions, esophageal stenosis, esophagectomy, colonic transplant, gastric tube

## Abstract

**Introduction:** Accidental caustic burns of the esophagus in children represent a significant global health challenge, often necessitating esophageal reconstruction. The aim of this study is to compare the efficacy and morbidity related to esophagus replacement with colonic and gastric tube transplants in a pediatric population followed for caustic stenosis. **Methods:** This retrospective study was conducted at a tertiary pediatric surgery unit for children treated from January 1989 to December 2022. We compared colonic and gastric tube esophageal replacement. Short term (within 30 days) and mid-term outcomes and complications were reviewed. Statistical evaluation was considered using a Chi-square test for categorical data analysis. **Results:** A total of 124 children with caustic esophageal burns were included. Among them, 23 (18.5%) had a gastric tube transplant for esophagus replacement and 101 (81.5%) a colonic transplant. During surgical intervention, we found a significantly higher risk of complications when using a colonic transplant (34%, *p* < 0.001). There was no significant statistical difference in postoperative short term and mid-term complications between the two techniques. Twenty-six (26%) of the children required a reoperation, with a higher risk in the gastric tube transplant group (*p* < 0.001). Endoscopic dilatation after surgery was also performed on a higher number of children who had received a gastric tube transplant (*p* = 0.005). Overall, 97.6% recovered full normal oral feeding. **Conclusions:** We found that colonic and gastric tube replacement are both good options for pediatric esophageal replacement after a caustic injury and show effectiveness over time. Gastric tube transplants carried a slightly higher risk of reoperations and a higher number of dilatations post-surgery. However, our groups are not really comparable, due to the much higher number of colonic transplants. Both surgical options have to be considered during surgery, and the choice depends on the anatomy of the patient. Our future research will focus on assessing long term quality of life and the potential risk of neoplastic complications.

## 1. Introduction

**Figure 1:**
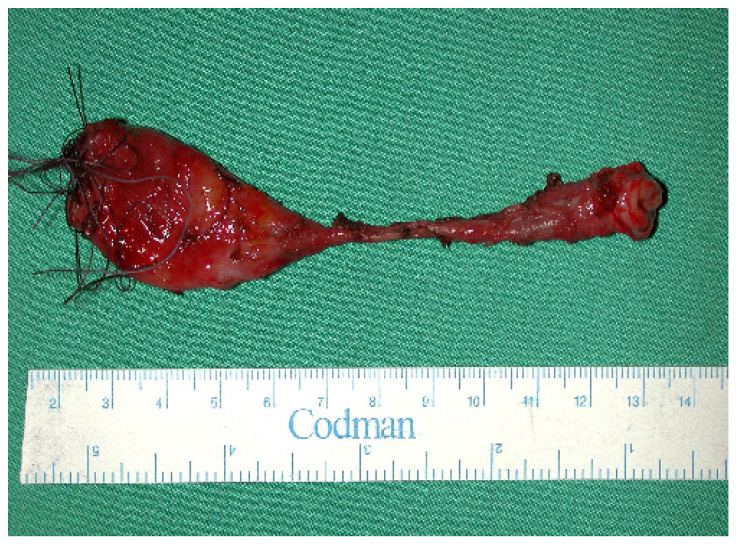
Showing multiple stenosis of the resected native esophagus.

Accidental caustic burns of the esophagus in children remain a global health problem. Conservative management by endoscopic dilatation over the first months may be possible for isolated short stenosis (1–2 cm). For more than 3 cm, for multiple stenosis, for those with a tracheoesophageal fistula, surgical treatment is mandatory (Figure 1). This decision has to be taken six months after the injury, as by then the lesions are totally stabilized [1,2].

A preoperative evaluation of the oropharynx and larynx must be done before surgery, as associated lesions are not unusual (15%) [3]. It should include the examination of vocal cord movements during spontaneous breathing under anesthesia, as the vocal cords will play an important role when eating during the recovery period.

There are several possibilities for esophageal replacement [4,5]. The colon is a bowel segment that offers the advantage of different vascular supplies and is long enough to choose the best segment and length needed for the replacement. This colon transposition will have no efficient propulsive contractions during eating and will be emptied by gravity. Nevertheless, Jones demonstrated in 1971 on animals, and then in humans, that an acid reflux in the transplant induces a contraction that may protect the colonic mucosa against acid aggression [6].

The gastric tube is another possibility as a substitute of the esophagus. A surgical procedure creates a tube along the long gastric curve. The gastrostomy previously performed along the great curvature to feed the child must be checked during the surgical procedure to be sure that the gastric vessels were not damaged, as they are essential to bring the blood to the gastric tube. In this procedure, anti-reflux wrap is not possible because of the lack of residual gastric tissue, which induces a postoperative gastroesophageal reflux.

Gastric pull-ups are also realized, involving mobilization of the entire stomach with one anastomosis in the neck for the continuity. Acid and/or biliary reflux is reported in about 30% of patients even without pyloroplasty, with a prevalence of reflux esophagitis ranging from 30% to 78% [7,8,9]. In small children, the procedure is known to compromise the lung function and the heart venous return [10,11].

Finally, the esophagus can be replaced by a segment of the small bowel with a vascularized flap. But due to its very short vascular disposition, a jejunal transplant requires a very long segment, not well adapted in its anatomical configuration with vascular arcades and which remains fragile and reactive to acidity [4,12]. Recent studies highlight the promising results of free jejunal and pedicled supercharged jejunal flaps for esophageal reconstruction, even in cases with severe epiglottic damage [13].

According to previous studies, there are many short and long term complications, such as leaks of the proximal anastomosis in the short term and restenosis in the longer term. The final aim is to allow the children to be fed and avoid failure to thrive and growth retardation. Nevertheless, many children experience noisy breathing and, coughing refluxes and acquire strange eating habits, for instance drinking between each bite.

The aim of this retrospective study was to review our short- and mid-term outcomes and compare results of the use of gastric or colic tube in pediatric esophageal replacement. We assessed complications related to surgical approach and encountered during the follow-up.

## 2. Materials and Methods

This single-center retrospective study was conducted in the Department of Pediatric Surgery of the University Hospital in Lausanne (Switzerland). It included children, under eighteen years old, victims of esophageal corrosive stenosis due to ingestion of caustic soda, operated and followed between January 1989 and December 2022. The study was approved by the local ethical committee (protocol CER-VD, BASEC-ID 2021-02460).

The medical records were reviewed for demographics, surgical details, complications (perioperative to mid-term), and outcomes.

Exclusion criteria included all patients referred secondary to one or more esophageal procedures, cases with a surgical indication other than caustic esophageal burn, children with incomplete follow-up records, and any patients with a file indicating a refusal to re-use data for scientific research.

Our partnership with a local team made it possible to treat and subsequently follow the children there. The Department of Pediatric Surgery of the University Hospital in Lausanne (CHUV) has been providing medical support to Benin and Togo since 1980 by organizing yearly surgical missions and follow-up of the operated children. Now, the Pediatric Hospital of Sedo-Goho in Abomey gathered the children, sorted them out according to their pathology, and provided the premises for consultations. Gastrostomies were performed at the hospital in Benin by local pediatric surgeons. During our common consultation in October, the history and radiological investigations of the children were discussed in order to determine a transfer to Switzerland if necessary. The goal of our cooperation was to treat the greatest possible number of children in their own country but transfer to Switzerland difficult cases, such as esophagus replacement.

**Figure 2:**
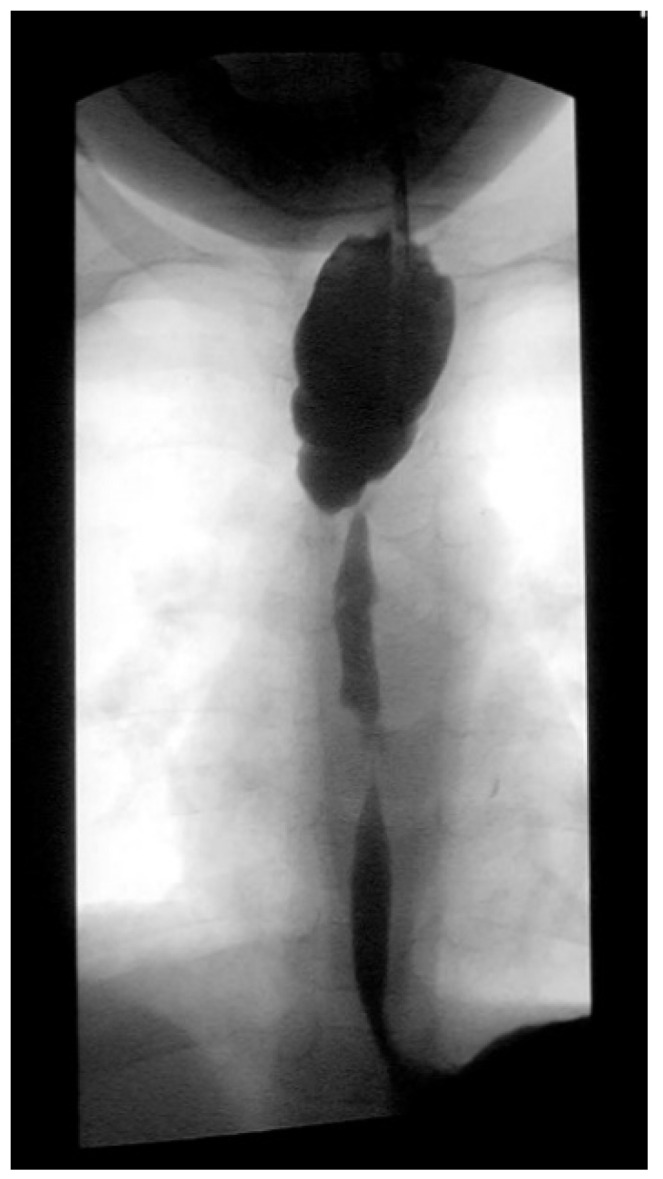
Preoperative esophagogram.

**Figure 3:**
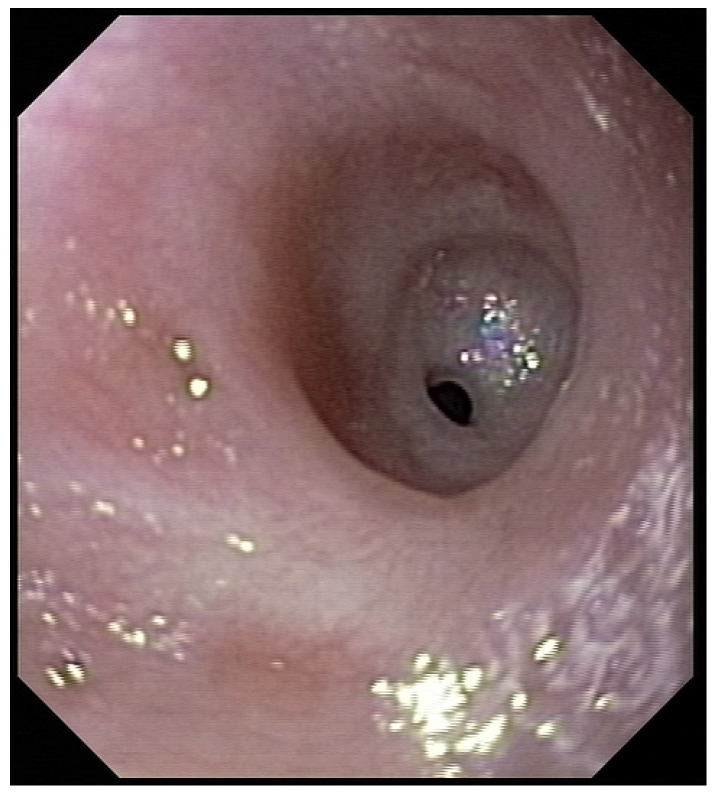
Preoperative endoscopic evaluation to assess the grade of esophageal stenosis.

Three weeks before surgery, the children were transferred from Africa to Switzerland for the evaluation of their nutrition status, as they eventually needed to be fed carefully and progressively. A thoracic X-ray and an esophagogram are performed (Figure 2). Intubation allowed an endoscopic evaluation of the grade of esophageal and pharyngeal stenosis and mobility of the vocal cords (Figure 3). This evaluation was done with our ear nose and throat (ENT) colleagues, and eventual tracheotomy was discussed for very high stenosis of the larynx without residual damage to the esophagus.

Surgery starts by positioning the child in a supine position for a laparoscopic approach. This positioning has to be precisely done by the surgeons. The first step is the esophagectomy, laparoscopically (since 2007) or manually, with closed chest [8]. Laparoscopic vision allows us to dissect under visual control all the esophagus up to the clavicle. Vagus nerves are carefully dissected. Pneumothorax, pulmonary veins sections, or left bronchi holes have to be avoided. The second part is the section of the esophagus after a cervical left incision and dissection of the tissue preserving the two recurrent laryngeal nerves. The third step is a medial laparotomy in order to remove the esophagus and prepare the transplant. The fourth part consists in choosing the transplant, depending on the vascular configuration, positioning of the gastrostomy, length of stomach, and length needed for the transplant.

**Figure 4:**
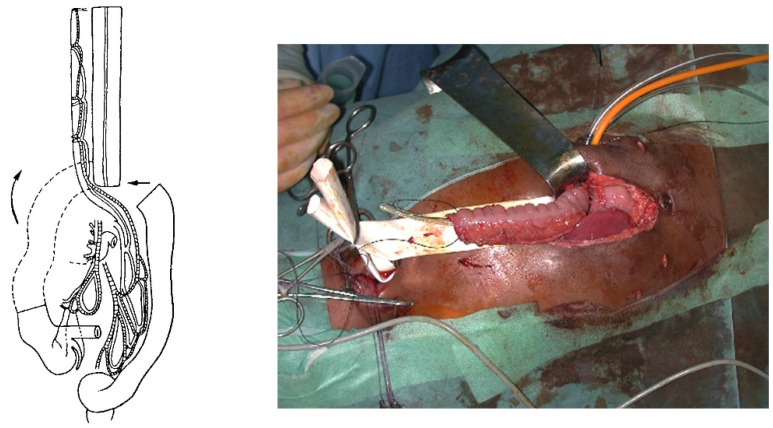
Isoperistaltic transverse colon vascularized by the left colic artery.

For a colon transplant, the dissection and preparation of the vascular vessels have to be carefully done after the measurement of its length. The transverse colon is prepared with its blood supply coming from the left or the middle colonic vessels according to the vascularization (Figure 4). The colon has to brought to the thoracic cavity behind the stomach, and the vessels have to be long enough without tension. The interposed colonic transplant is placed in the mediastinum in the isoperistaltic position.

**Figure 5:**
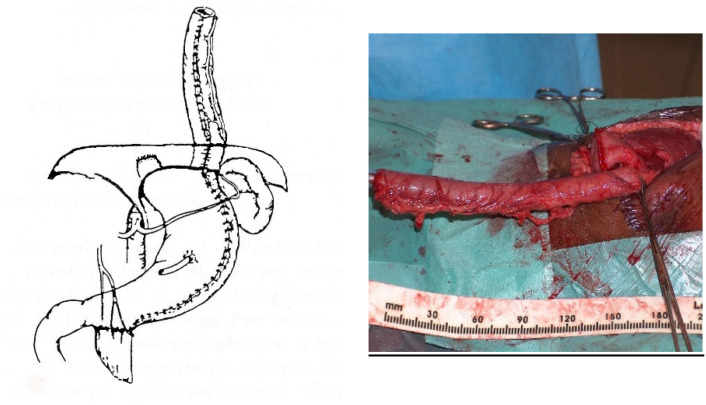
Reversed gastric tube vascularized by the left gastroepiploic artery.

**Figure 6:**
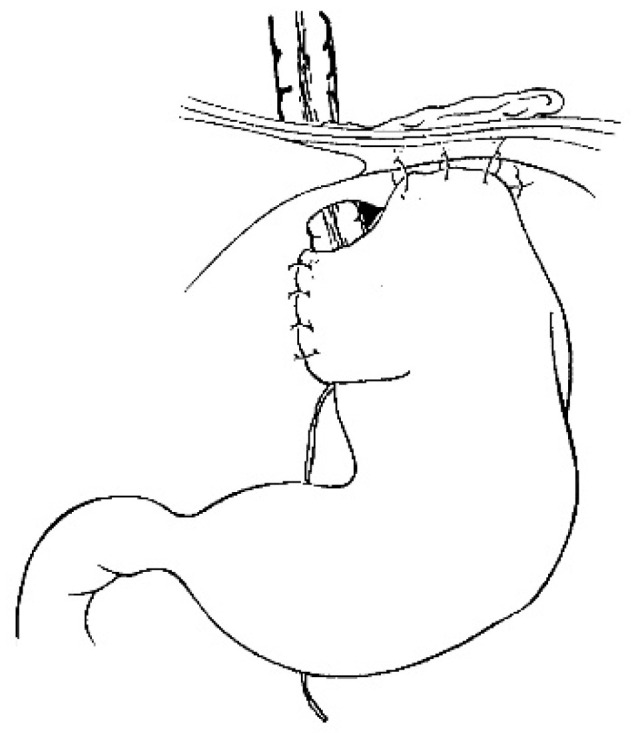
The anti-reflux wrap for colonic transplant [14].

For a gastric tube, the left and right gastric vessels have to be preserved in order to prepare the long curve; we use a gastrointestinal stapler three or four times to prepare and create the gastric tube for an antiperistaltic position (Figure 5). The final steps are the anastomosis of the colon with an end-to-end anastomosis, the confection of a gastrostomy if not existing, and the anastomosis of the transplant on the stomach and to the proximal esophagus if existing or higher in the pharynx; an anti-reflux is created at the end of the operation only after a colon transplant [7]. This anterior wrap covers 3 cm of the distal transplant, similar to the Dor’s procedure but sutured to the anterior aspect of the right crus of the diaphragm instead of to the esophageal wall (Figure 6). The wrap needs to be loose enough not to compress the vascular pedicle located behind the transplant. The opening of the hiatus behind the transplant is never closed [14].

The children are routinely fitted with a naso-transplant catheter, and through the gastrostomy they receive a gastro-transplant catheter, a gastric catheter, and a gastrojejunal catheter for feeding. An *endless wire* is also inserted from the gastrostomy to the nose, and from the nose to the gastrostomy, to allow for any future safe esophageal dilatation.

The children are extubated as soon as possible after the surgery. During 10 days, a gastric suction is maintained and the children are fed through the gastrojejunal catheter. About ten days after surgery, an esophagogram is performed to exclude any leak and start oral feeding. If cervical anastomotic leakage is present, conservative treatment with esophageal suction is mandatory for one week, and oral feeding starts seven days later. Otherwise, oral feeding starts immediately, and enteral feeding progressively decreases and is stopped. Feeding through the gastrostomy catheter is used to complete the oral feeding, which is usually difficult at the beginning as the child has to adapt to the new condition of eating by mouth. Once a normal diet is reached and the weight stabled, the gastrostomy catheter is removed. A routine endoscopic examination is done in the course of the following two months. A second esophagogram is normally done at two months postoperatively.

Complications are defined as “early” when they occur within 30 days after surgery, and as “mid-term” when they occur after 30 days.

Statistical evaluation was done using a Chi-square test. Significance was set as *p* < 0.05.

## 3. Results

Between January 1989 and December 2022, 145 children were operated in our hospital for esophageal replacement. All the children came from West Africa, other African countries, and Europa. Twenty-one patients were excluded from the study based on our exclusion criteria, due to secondary transfer of patients with failed esophagoplasty or when the patient records were incomplete.

One hundred and twenty-four (124 files of children were considered in the study, with a distribution of 52% boys and 48% girls (ratio 1.1:1). Ninety-three percent of them came from West Africa, 5% from other Africa countries, and 2% from Europa. The average preoperative weight was below percentile three in 53% of the children.

At the time of the accident, the mean age was 37 months (from three to 148 months) and the average time spent until the intervention was 31 months (from seven to 124 months). The mean age of children operated was 70 months (from 18 to 187 months). The average total length of follow-up in Switzerland was four months (from one to 24 months) (Table 1).

The preoperative evaluation showed that, of the 21 (17%) children with pharyngeal injuries, three children had vocal cord injury and six children had a preoperative tracheotomy. Nineteen (15%) were operated with the collaboration of our pediatric otolaryngology colleagues in order to dissect the pharyngeal walls with a high pharyngeal anastomosis of the colon. Total feeding by the gastrostomy was present preoperatively in 85.5% of our patients, of whom 74% had a complete dysphagia.

All the operations were performed by the same senior surgeon from 1989 to 2014 in a one-stage closed-chest esophagectomy procedure. Laparoscopical esophagestomy had been realized since 2007. From 2014 to 2022, the interventions were carried out by two senior surgeons using the same surgical technique.

Fifty-eight (47%) of our patients underwent a transhiatal esophagectomy under laparoscopic control: 46 children (46/101; 46%) in the colonic group; and 12 children (12/23; 52%) in the gastric group. The average operative time was shorter among those who underwent a gastric replacement (461 min vs. 530 min, *p* = 0.03) (Table 2).

Sixty-six of our patients underwent an esophagectomy after a laparotomy and blind dissection with closed chest.

The colon was in an isoperistaltic position in 98 cases and antiperistaltic in three patients. Intraoperative complications were recorded in 37 (30%) children, with a significant difference between both groups (34% for colon replacement against 13% for gastric replacement, *p* = < 0.001; OR 26.0) (Table 3). The most frequent complications were: pneumothorax (*n* = 9); vascular injury (*n* = 9) concerning the aorta (*n* = 2), left internal carotid artery (*n* = 1), left internal jugular vein (*n* = 1), left bronchial artery (*n* = 1), transplant pedicle (*n* = 1), and injury to arteries not described (*n* = 3); airway injury (*n* = 6) involving the trachea (*n* = 4) and the left main bronchus (*n* = 2); and finally neurological injury (*n* = 5) due to traction or sections of the left recurrent nerve (*n* = 4) or left phrenic nerve (*n* = 1).

The median stay in the intensive care unit was five days (from one day to 30 days). The average period of recovery in our surgical pediatric unit was 22 days (from 11 days to 82 days), with 23 and 21 days respectively for colonic and gastric transplants.

For early postoperative complications, there are a total of 125 complications in 75 (60%) children within the first 30 days (Table 4). There was no difference between the two groups. The most common complications were cervical leaks in 15% of the children, with 100% of spontaneous resolution after the prolongation of a naso-transplant aspiration. With one esophageal anastomosis for the gastric transplant compared with two for the colonic, the latter appears to present a significant increase in cervical leakage (9% vs. 16%, *p* = 0.001; OR 10.9) (Table 5). This was followed by anemia and pneumothorax, each occurring in 14% of cases, eating disorders in 11%, pneumonia and infections each in 10%, and pyloric spasm in 7%. Among children with an eating disorder, three (2%) cases were checked by our ENT colleagues because of repetitive tracheal false routes. Abscesses occurred in 4% of cases and were exclusively localized to the abdominal wall. Complications are summarized in Table 5.

Unplanned reoperations related to the initial operations concerned 32 (26%) children, with a significant difference between both techniques (25% for colon replacement and 30% for gastric replacement; *p* = 0.001) (Table 6). Indications were exploratory laparotomy in ten (8%) children for abdominal adhesions, occlusive syndrome, revision of the transplant anastomosis, and revision of the anti-reflux wrap; then, ENT revision in 10 (8%) children because of recurrent pharyngeal stenoses, who were treated with laser microlaryngoscopy. Other patients with respiratory issues received a tracheotomy or underwent surgical revision of a previous tracheotomy. Pyloroplasty was performed in three (2%) children for long-term pylorospasm (more than six weeks). A total of 15 (12%) children were reoperated within the 30 days and 22 (17%) after 30 days. Additionally, 11 (9%) children required multiple operation during their follow-up. Of these, nine cases were due to ENT revision.

Concerning mid-postoperative complications, more than 30 days after surgery we found 90 complications in 55 (44%) children (39% vs. 46%; *p* = 0.124) (Table 7). This rate is essentially due to gastroesophageal reflux disease. Eating disorders remained a complication in 10 (8%) patients, with 7% of children requiring an evaluation by our ENT colleagues. Pyloric spasms occurred in 7 (6%) patients.

For the development of anastomosis stenosis, endoscopic dilatation was required for 55 (44%) of patients and was significantly higher in patients having undergone gastric tube transplant (74% vs. 38%; *p* = 0.005; OR 8.0) (Table 8). The indications were symptomatic eating/swallowing difficulties or significant stenosis during the endoscopy, or both. They were performed on average from the second postoperative month (from 17 days to 329 days postoperative), with an average of three endoscopies per child (from one to 20 endoscopies).

Full oral feeding was achieved in 98.4% of children. The average time to achieve a normal diet was 50 days, 49 days for colonic transplants, and 56 days for gastric transplants. But three patients never recovered normal oral feeding despite iterative dilatations, and required a closed follow-up with an orthophonist and a nutritionist, and a specific diet plan.

## 4. Discussion

This study shows no significant difference within 30 days in the incidence of complications after esophageal replacement when comparing the use of either a colon transplant or a gastric tube. This is also our conclusion for our longer follow-up period. Nevertheless, morbidity after this very demanding surgery remains high and requires careful follow-up during a few months after surgery.

In 1907, Professor Cesar Roux performed the first successful esophageal replacement (so-called esophagoplasty) on a 12-year-old child in Lausanne, Switzerland [15]. The child suffered from caustic stenosis. Since then, many surgical procedures have been used to replace the esophagus. After having practiced for 24 years (1966–1989) two-stage esophagoplasties using retrosternal colonic transplants during, we introduced the one-stage procedure in 1989, placing the transplant in the posterior mediastinum, following a closed-chest esophagectomy [16,17].

In 2007, Professor Olivier Reinberg developed the safe dissection of the esophagus by laparoscopy [7,18]. Also, during this period from 1989 to 2007, esophagectomies were performed following a laparotomy and blind dissection with closed chest [18]. This approach led to 16 of the 37 intraoperative complications encountered, with a higher proportion of arterial lesions. Thus, we decided not to separate these patients into different groups, as laparoscopy was able to enhance gains in safety without compromising results [18].

As in many other pediatric surgical departments, isoperistaltic colonic transplants are our treatment of choice for esophageal replacements [4,12]. Reversed gastric tubes were used only if colonic transplants were technically not feasible. This choice of treatment was supported by our experience and publications, which demonstrated that these colonic replacements had either the lowest rate of major postoperative complications or a superior overall satisfaction rate when compared to gastric in children [7,19,20,21,22].

From 1966 to 1989, we used to perform the lower anastomosis according to various techniques (Sherman’s, Belsey’s, and Waterston’s) [14,17]. All were followed by a high rate of reflux in the colonic transplant, especially when the Waterston technique was used [14]. With the introduction of the one-stage procedure in 1989, almost all the lower anastomosis was made with Sherman’s technique [23]. Because the two first cases operated with this new approach had huge reflux in the transplant, we added an anti-reflux procedure [14].

Such operations are not exempt from intraoperative complications, especially in the case of colonic surgery. Our results highlight the complexity of a colonic transplant compared to a gastric transplant, which is associated with significantly higher intraoperative complications and longer operating times. Other comparative studies also seem to demonstrate a reduction in intraoperative complications and operating time using the gastric transplant [24].

Concerning early term complications (≤30 days), almost 2/3 of children experienced problems regardless of the technique used. These values seem to be comparable to other studies [4,25,26]. The largest series of comparisons between gastric and colon transposition validate our results, showing no significant difference in early or late complications [12]. The most common complication encountered was cervical leakage (15%). It seemed to be significantly even more prevalent in colonic tubes (16%; *p* = 0.001) with a rate comparable to the average of published studies [4,27]. But in comparison with other studies, our patients with reversed gastric tube interposition presented less cervical leakage [28,29,30].

A strong experience in operative techniques does not rule out morbidities requiring unplanned reoperations. In our study, such reoperations were necessary for 26% of our patients. The literature focusing on the outcomes of reoperations following esophageal reconstruction in pediatric patients after caustic ingestion is poor. However, our findings indicate a higher incidence of unplanned reoperation than the 17.2% reported in the study by Bludevich et al. [26]. This can be explained by the inclusion of patients more complex with high anastomoses, who required multiple laryngopharyngeal revision. If these patients were excluded of our sample, the rate of unplanned reoperations would fall to 18%. These unplanned reoperations appear in our results to be significantly more frequent among the gastric tube transplant group (30% vs. 25%; *p* = < 0.001), although the literature suggests that colonic transplants may carry a higher risk of complications that could lead to unplanned reoperations. None of the reoperations directly involved the transplant viability.

Three patients in the colic transposition group presented with repetitive episodes of vomiting and delayed gastric emptying for more than six weeks postoperatively. These patients subsequently underwent a Heineke-Mikulicz pyloroplasty. Unlike other studies involving larger patient and extended follow-ups, we do not routinely perform this operation even if damage to the vagus nerve was suspected or objectivized [5,12,19,31,32,33].

Concerning mid-term complications, after one month, 44% of the children showed unresolved problems. Reflux has been our main complication, with a significant incidence in colic transplant (*p* = < 0.001). Since 1989 we have been using a new anti-reflux wrap (ARW) in colic transplant, employing an anterior wrap similar to Dor’s but fixed to the right crus [14]. It could be potentially life threatening when gastric reflux erodes the wall and leads to massive bleeding without previous pain in an insensitive transplant. Hamza reported no reflux after his transhiatal procedure, but he systematically associates an anti-reflux wrap (not described), a vagotomy, and a pyloroplasty, without evidencing a pre-existing gastrointestinal reflux (GER) [21,31]. A second esophagogram performed about six weeks after surgery is a better way to identify a gastrocolonic reflux that could become a clinical issue. If not routinely done, it must be prescribed in all patients who present symptoms of reflux, such as difficulties in eating, vomiting, or chronic cough etc. On the second esophagogram, the rate of stasis in the transplant is almost the same in both groups. One patient in four presented a late stasis in the transplant, but this result may be overestimated. The radiologists who performed the examinations were not always aware of the poor motility of colonic transplant compared to a normal esophagus. An absence of esophageal motility and a delay in clearing the transplant could improperly be diagnosed as stasis.

Regarding the risk of anastomotic stenosis, we performed standardized endoscopic controls of the anastomosis after the second month. Endoscopic dilatation was performed only if the patients suffered from persistent dysphagia, or if the diameter of the esophageal anastomosis was judged to be too narrow during the endoscopy. In our sample, the gastric transplant group had a significantly greater need for dilatation (74% vs. 38%; *p* = 0.005). Stenotic recurrences generally occur around two to three months postoperatively, or even several years later. Clinical follow-up and endoscopic dilatations are carried out on the patients in their own country. This follow-up care is crucial to monitor for complications and ensure the success of the flap. Full oral nutrition was restored in 97.6% (121/124) children. On average, they achieved a normal diet at 50 and 49 days postoperatively for colonic and gastric transplant, respectively. They were all managed by a multidisciplinary team that included nutritionists to ensure optimal intake after surgery. This led to improved percentile weight in 20% of the children, as shown in our mid-term follow-up.

The operation was unsuccessful in two colonic transplants and one gastric transplant. One of the colonic transplant failures may be explained by the discovery of esophageal tuberculosis in the postoperative period, which was difficult to treat and where multiple dilatations did not resolve the stenosing inflammatory process. The two other failures were probably due to the complexity of the preoperative oropharyngeal lesions. Indeed, the difficulty of esophageal replacement is further complicated when the patient presents this type of lesions. It is essential to identify these lesions prior to any definitive intervention, in order to avoid any resulting functional failure. The long-term outcomes in patients with pharyngeal strictures continue to be poor. In our study, these children presented with a high proportion of multiple surgical revision and endoscopic dilatations by our otolaryngology colleagues.

These patients are indeed at risk of long-term neoplastic complications. The incidence of esophageal carcinoma may be significantly higher in these patients, ranging from 2–30% [1,34,35,36]. Long-term dysplasia screening is necessary for these patients to allow early detection of neoplasia.

The overall mortality rate varies, ranging from 0% to 10% in various studies [4,10,21,37]. We had no reported deaths among our patients.

Working with developing countries contributes to an exchange of interests, and we can learn from them as much as they can learn from us. Medical cooperations cannot be limited to completing operations and then leaving. The best organizations are those that integrate the local socio-medical community by working together with physicians, nurses, and social workers in a personal and collaborative manner. The aim is to share our professional experience with local medical teams, so that they can then organize their own work and pursue it on their own. The foreign organization will continue to support them, in particular by donating the medical equipment that will allow these professionals to perform independently.

## 5. Conclusions

In the short- and mid-term, both the colonic transplant and the gastric tube prove to be adequately efficient and safe for replacing the esophagus in children with caustic stenosis. Although gastric tube transplants carried a slightly higher risk of reoperations and required a greater number of post-surgical dilatations, both techniques successfully restore the ability to swallow. This surgery and its follow-up have proved feasible on patients from countries with resource-limited settings. Determining the long-term quality of life for these patients is now essential.

## Figures and Tables

**Table 1:** General characteristics.

	All Patients (*n* = 124)	Colic Transplant (*n* = 101)	Gastric Transplant (*n* = 23)
Age at accident	37	37	37
Age at surgery	70	69	74
Time until the intervention	31		
Length of follow-up in Switzerland	4	4	5

Data are expressed as mean values in months.

**Table 2:** Length of interventions.

	All Patients (*n* = 124)	Colic Transplant (*n* = 101)	Gastric Transplant (*n* = 23)	*p*-Value
Set-up time	37	36	39	0.73
Laparoscopy technique surgery	515	530	461	0.03
Without laparoscopy technique surgery ^(a)^	368	370	346	0.37
Surgery independent of technique	435	443	410	0.26
Overall length of the procedure ^(b)^	552	560	522	0.25
laparoscopy technique	643	659	584	0.03
without laparoscopy technique ^(a)^	474	477	455	0.47

Data are expressed as means values in minute, ^(a)^ blind dissection with closed chest, ^(b)^ Time from start of anaesthesia to end of anaesthesia.

**Table 3:** Intraoperative complications.

	All Patients (*n* = 124)	Colic Transplant (*n* = 101)	Gastric Transplant (*n* = 23)	*p*-Value	OR (95% CI)
Intraoperative complication	37 (30%)	34 (34%)	3 (13%)	<0.001	26.0
splenectomy	1	1	0	0.32	1
splenic injury	1	1	0	0.32	1
intestinal perforation	3	3	0	0.08	3
necrosis	1	0	1	0.32	1
iatrogenic urethral injury	1	1	0	0.32	1
pneumothorax	9	9	0	0.003	9
airway injury	6	5	1	0.10	2.7
cardio-pulmonary system failure	3	3	0	0.08	3
vascular injury	9	8	1	0.02	5.4
neurological injury	5	5	0	0.03	5.0

**Table 4:** Postoperative complications.

	All Patients (*n* = 124)	Colic Transplant (*n* = 101)	Gastric Transplant (*n* = 23)	*p*-Value	OR (95% CI)
≤30 days of surgery	75 (60%)	62 (61%)	13 (57%)	0.39	0.7
>30 days after surgery	55 (44%)	46 (46%)	9 (39%)	0.12	2.4

**Table 5:** Complications within ≤30 days of surgery.

	All Patients (*n* = 124)	Colic Transplant (*n* = 101)	Gastric Transplant (*n* = 23)	*p*-Value	OR (95% CI)
Cervical leak	18 (15%)	16 (16%)	2 (9%)	0.001	10.9
*Pulmonary*					
pneumothorax	17 (14%)	13 (13%)	4 (17%)	0.03	4.8
pneumonia	12 (10%)	9 (9%)	3 (13%)	0.08	3
*Cardio-vascular*					
iatrogenic tamponade	2 (2%)	2 (2%)	0	0.16	2
cardiorespiratory arrest	1 (1%)	1 (1%)	0	0.32	1
heart rythm disorder	2 (2%)	2 (2%)	0	0.16	2
pneumopericardium	1 (1%)	1 (1%)	0	0.32	1
*Abdominal*					
pyloric spasm	9 (7%)	7 (7%)	2 (9%)	0.10	2.8
pneumoperitoneum	1 (1%)	1 (1%)	0	0.32	1
ileus	4 (3%)	3 (3%)	1 (4%)	0.32	1
splenic infarct	1 (1%)	1 (1%)	0	0.32	1
ENT check	3 (2%)	2 (2%)	1 (4%)	0.56	0.3
Eating disorder	14 (11%)	9 (9%)	5 (22%)	0.29	1.1
Sepsis	3 (2%)	3 (3%)	0	0.08	3
Abscess	5 (4%)	5 (5%)	0	0.03	5
Infection	12 (10%)	10 (10%)	2 (9%)	0.02	5.3
Anemia	17 (14%)	15 (15%)	2 (2%)	0.002	9.9
Malaria crisis	2 (2%)	2 (2%)	0	0.16	2
Chylothorax	3 (2%)	2 (2%)	1 (4%)	0.56	0.3
Extrapyramidal syndrome	4 (3%)	4 (4%)	0	0.05	4
Delirium	1 (1%)	0	1 (4%)	0.32	1
Horner syndrome	1 (1%)	0	1 (4%)	0.32	1

ENT: Ear nose and throat.

**Table 6:** Unplanned reoperations.

	All Patients (*n* = 124)	Colic Transplant (*n* = 101)	Gastric Transplant (*n* = 23)	*p*-Value	OR (95% CI)
Reoperation	32 (26%)	25 (25%)	7 (30%)	0.001	10.125
*Type of reoperation*					
pleural fluid puncture	1 (1%)	0	1 (4%)	0.32	1
bronchoscopy	3 (2%)	2 (2%)	1 (4%)	0.56	0.3
pericardial drain	2 (2%)	2 (2%)	0	0.16	2
exploratory laparotomy	10 (8%)	7 (7%)	3 (13%)	0.21	1.6
pyloroplasty	3 (2%)	3 (3%)	0	0.08	3
repair/suppression of anti-reflux valve	3 (2%)	3 (3%)	0	0.08	3
cure of abdominal eventration	1 (1%)	1 (1%)	0	0.32	1
ENT multiple revision	10 (8%)	9 (9%)	1 (4%)	0.01	6.4
material issue	4 (3%)	3 (3%)	1 (4%)	0.32	1
chest tube placement	4 (3%)	3 (3%)	1 (4%)	0.32	1
abscess excision	3 (2%)	3 (3%)	0	0.08	3

ENT: Ear nose and throat.

**Table 7:** Complications after 30 days of surgery.

	All Patients (*n* = 124)	Colic Transplant (*n* = 101)	Gastric Transplant (*n* = 23)	*p*-Value	OR (95% CI)
*Pulmonary*					
pneumonia	5 (4%)	5 (5%)	0	0.03	5
*Abdominal*					
pyloric spasm	7 (6%)	6 (6%)	1 (4%)	0.06	3.6
abdominal eventration	1 (1%)	1 (1%)	0	0.32	1
ileus	6 (5%)	4 (4%)	2 (9%)	0.41	0.7
gastric ulcer	1 (1%)	1 (1%)	0	0.32	1
gastric dyskinesia	1 (1%)	0	1 (4%)	0.32	1
eperon gastric	1 (1%)	0	1 (4%)	0.32	1
GERD	33 (27%)	31 (31%)	2 (9%)	<0.001	25.5
iterative haematemesis	1 (1%)	1 (1%)	0	0.32	1
ENT check	9 (7%)	9 (9%)	0	0.003	9
Eating disorder	10 (8%)	8 (8%)	2 (9%)	0.06	3.6
Sepsis	1 (1%)	1 (1%)	0	0.32	1
Abscess	7 (6%)	7 (7%)	0	0.01	7
Infection	5 (4%)	4 (4%)	1 (4%)	0.18	1.8
Esophagitis	1 (1%)	0	1 (4%)	0.32	1
Subcutaneous emphysema	1 (1%)	1 (1%)	0	0.32	1

ENT: Ear nose and throat, GERD: Gastroesophageal reflux disease.

**Table 8:** Dilatations.

	All Patients (*n* = 124)	Colic Transplant (*n* = 101)	Gastric Transplant (*n* = 23)	*p*-Value	OR (95% CI)
Endoscopic dilatation	55 (44%)	38 (38%)	17 (74%)	0.01	8.02
Number of dilatation ^(a)^	3	3	2	0.66	0.2
Time to 1st dilation (days) ^(a)^	61	64	55	0.41	0.68
Recurrence of stenosis ^(a)^	3	1	2	0.56	0.33

^(a)^ Data are expressed as means values.

## Data Availability

The data presented in this study are available on request from the corresponding author. The data are not publicly available.

## References

[1] Contini S., Scarpignato C. (2013). Caustic injury of the upper gastrointestinal tract: A comprehensive review. World J. Gastroenterol..

[2] Panieri E., Rode H., Millar A.J.W., Cywes S. (1998). Oesophageal replacement in the management of corrosive strictures: When is surgery indicated?. Pediatr. Surg. Int..

[3] Reinberg O. (2014). Les oesophagoplasties chez l’enfant. EMem. Acad. Natl. Chir..

[4] Chirica M., Bonavina L., Kelly M.D., Sarfati E., Cattan P. (2017). Caustic ingestion. Lancet.

[5] Spitz L. (2014). Esophageal replacement: Overcoming the need. J. Pediatr. Surg..

[6] Chirica M., Bonavina L., Kelly M.D., Sarfati E., Cattan P. (1971). Functional evaluation of esophageal reconstructions. Ann. Thorac. Surg..

[7] Reinberg O. (2016). Esophageal replacements in children. Ann. N. Y. Acad. Sci..

[8] Reinberg O. (2008). Laparoscopic esophagectomy in esophageal replacements in children. J. Laparoendosc. Adv. Surg. Tech. A.

[9] Gust L., Ouattara M., Coosemans W., Nafteux P., Thomas P.A., D’Journo X.B. (2016). European perspective in Thoracic surgery-eso-coloplasty: When and how?. J. Thorac. Dis..

[10] Awad K., Jaffray B. (2017). Oesophageal replacement with stomach: A personal series and review of published experience. J. Paediatr. Child Health.

[11] Spitz L. (2009). Gastric transposition in children. Semin. Pediatr. Surg..

[12] Arul G.S., Parikh D. (2008). Oesophageal replacement in children. Ann. R. Coll. Surg. Engl..

[13] Aksoyler D., Ercan A., Losco L., Chen S., Chen H. (2022). Experience in reconstruction of esophagus, epiglottis, and upper trachea due to caustic injuries in pediatric patients and establishment of algorithm. Microsurgery.

[14] Maurer S.V., Estremadoyro V., Reinberg O. (2011). Evaluation of an antireflux procedure for colonic interposition in pediatric esophageal replacements. J. Pediatr. Surg..

[15] Roux C. (1907). L’oesophago-jéjuno-gastrostomose, nouvelle opération pour rétrécissement infranchissable de l’oesophage. Sem. Méd..

[16] Reinberg O. (1989). Les Oesophagoplasties Chez L’enfant. Master’s Thesis.

[17] Reinberg O., Genton N. (1997). Esophageal replacement in children: Evaluation of the one-stage procedure with colic transplants. Eur. J. Pediatr. Surg..

[18] Maurer S.V., Roessingh A.d.B., Reinberg O. (2013). Comparison of transhiatal laparoscopy versus blind closed-chest cervicotomy and laparotomy for esophagectomy in children. J. Pediatr. Surg..

[19] Burgos L., Barrena S., Andrés A.M., Martínez L., Hernández F., Olivares P., Lassaletta L., Tovar J.A. (2010). Colonic interposition for esophageal replacement in children remains a good choice: 33-year median follow-up of 65 patients. J. Pediatr. Surg..

[20] Tannuri U., Maksoud-Filho J.G., Tannuri A.C.A., Andrade W., Maksoud J.G. (2007). Which is better for esophageal substitution in children, esophagocoloplasty or gastric transposition? A 27-year experience of a single center. J. Pediatr. Surg..

[21] Hamza A.F., Abdelhay S., Sherif H., Hasan T., Soliman H., Kabesh A., Bassiouny I., Bahnassy A.F. (2003). Caustic esophageal strictures in children: 30 years’ experience. J. Pediatr. Surg..

[22] Yildirim S., Köksal H., Celayir F., Erdem L., Oner M., Baykan A. (2004). Colonic interposition vs. gastric pull-up after total esophagectomy. J. Gastrointest. Surg..

[23] Sherman C.D., Waterston D. (1957). Oesophageal reconstruction in children using intrathoracic colon. Arch. Dis. Child.

[24] Javed A., Pal S., Dash N.R., Sahni P., Chattopadhyay T.K. (2011). Outcome Following Surgical Management of Corrosive Strictures of the Esophagus. Ann. Surg..

[25] Coopman S., Michaud L., Halna-Tamine M., Bonnevalle M., Bourgois B., Turck D., Gottrand F. (2008). Long-term Outcome of Colon Interposition After Esophagectomy in Children. J. Pediatr. Gastroenterol. Nutr..

[26] Bludevich B.M., Kauffman J.D., Smithers C.J., Danielson P.D., Chandler N.M. (2020). 30-Day Outcomes Following Esophageal Replacement in Children: A National Surgical Quality Improvement Project Pediatric Analysis. J. Surg. Res..

[27] Hunter C.J., Petrosyan M., Connelly M.E., Ford H.R., Nguyen N.X. (2009). Repair of long-gap esophageal atresia: Gastric conduits may improve outcome—A 20-year single center experience. Pediatr. Surg. Int..

[28] Randolph J.G. (1996). The reversed gastric tube for esophageal replacement in children. Pediatr. Surg. Int..

[29] Ein S.H. (1998). Gastric tubes in children with caustic esophageal injury: A 32-year review. J. Pediatr. Surg..

[30] Spitz L., Kiely E., Pierro A. (2004). Gastric transposition in children—A 21-year experience. J. Pediatr. Surg..

[31] Hamza A.F. (2009). Colonic replacement in cases of esophageal atresia. Semin. Pediatr. Surg..

[32] Sloan K., Morandi A., Lakshminarayanan B., Cox S.G., Millar A.J.W., Numanoglu A., Lakhoo K., Bradshaw C.J. (2018). Outcomes of Esophageal Replacement: Gastric Pull-Up and Colonic Interposition Procedures. Eur. J. Pediatr. Surg..

[33] Saleem M., Iqbal A., Ather U., Haider N., Talat N., Hashim I., Mirza M.B., Butt J., Mahmud H., Majeed F. (2020). 14 Years’ experience of esophageal replacement surgeries. Pediatr. Surg. Int..

[34] Lindahl H., Rintala R., Sariola H., Louhimo I. (1990). Cervical Barrett’s esophagus: A common complication of gastric tube reconstruction. J. Pediatr. Surg..

[35] Kiviranta U.K. (1952). Corrosion Carcinoma of the Esophagus 381 Cases of Corrosion and Nine Cases of Corrosion Carcinoma. Acta Oto-Laryngol..

[36] Kay M., Wyllie R. (2009). Caustic ingestions in children. Curr. Opin. Pediatr..

[37] Garritano S., Irino T., Scandavini C.M., Tsekrekos A., Lundell L., Rouvelas I. (2017). Long-term functional outcomes after replacement of the esophagus in pediatric patients: A systematic literature review. J. Pediatr. Surg..

